# Zoonotic Leprosy in the Southeastern United States

**DOI:** 10.3201/eid2112.150501

**Published:** 2015-12

**Authors:** Rahul Sharma, Pushpendra Singh, W.J. Loughry, J. Mitchell Lockhart, W. Barry Inman, Malcolm S. Duthie, Maria T. Pena, Luis A. Marcos, David M. Scollard, Stewart T. Cole, Richard W. Truman

**Affiliations:** National Hansen’s Disease Program, Baton Rouge, Louisiana, USA (R. Sharma, P. Singh, M.T. Pena, D.M. Scollard, R.W. Truman);; Louisiana State University School of Veterinary Medicine, Baton Rouge (R. Sharma, P. Singh, M.T. Pena);; Ecole Polytechnique Federale de Lausanne, Lausanne, Switzerland (P. Singh, S.T. Cole);; Valdosta State University, Valdosta, Georgia, USA (W.J. Loughry, J.M. Lockhart);; Florida Department of Health, Merritt Island, Florida, USA (W.B. Inman);; Infectious Disease Research Institute, Seattle, Washington, USA (M.S. Duthie);; Hattiesburg Clinic, Hattiesburg, Mississippi, USA (L.A. Marcos)

**Keywords:** leprosy, zoonotic leprosy, Hansen’s disease, Hansen disease, Mycobacterium leprae, bacteria, armadillos, nine-banded armadillo, Dasypus novemcinctus, zoonoses, epidemiology, biomarkers, genotype, southeastern United States

## Abstract

Nine-banded armadillos (*Dasypus novemcinctus*) are naturally infected with *Mycobacterium leprae* and have been implicated in zoonotic transmission of leprosy. Early studies found this disease mainly in Texas and Louisiana, but armadillos in the southeastern United States appeared to be free of infection. We screened 645 armadillos from 8 locations in the southeastern United States not known to harbor enzootic leprosy for *M. leprae* DNA and antibodies. We found *M. leprae*–infected armadillos at each location, and 106 (16.4%) animals had serologic/PCR evidence of infection. Using single-nucleotide polymorphism variable number tandem repeat genotyping/genome sequencing, we detected *M. leprae* genotype 3I-2-v1 among 35 armadillos. Seven armadillos harbored a newly identified genotype (3I-2-v15). In comparison, 52 human patients from the same region were infected with 31 *M. leprae* types. However, 42.3% (22/52) of patients were infected with 1 of the 2 *M. leprae* genotype strains associated with armadillos. The geographic range and complexity of zoonotic leprosy is expanding.

## Abstract 2

The geographic range and complexity of this disease are increasing.

Leprosy (Hansen disease), a chronic infectious disease caused by *Mycobacterium leprae*, primarily affects the peripheral nervous system and involves skin and other tissues (*1*). Although this disease is generally a rare disorder that occurs mainly in tropical and semitropical areas, the World Health Organization recorded 219,075 new leprosy cases globally in 2011, and 439,670 new cases were reported in the Western Hemisphere over the past decade (*2*,*3*). Although leprosy is curable by antimicrobial drug therapy, the treatment interval for this disease can require >2 years to complete, and underlying nerve damage caused by the infection might be irreversible. There are no established laboratory screening tests to detect leprosy; the disease must be diagnosed clinically. Therefore, physician awareness about leprosy and knowledge of populations potentially at risk for the infection, are paramount for early detection and treatment (*1*).

Leprosy was not present in the New World during pre-Columbian times and appears to have been introduced to the Western Hemisphere after colonization. Early case reports suggest the disease was well established in most countries surrounding the Gulf of Mexico by the 1750s (*4*,*5*). Genomic polymorphisms enable us to trace the spread of the disease worldwide and confirm the regional origins of most isolates (*6*). The disease is rare in the United States; only ≈13,000 cases have been recorded since the 1890s, and ≈200 new cases are reported each year. Most of these case-patients lived or worked outside the country in disease-endemic areas and might have acquired their disease abroad (*7*). However, approximately one third of all case-patients in the United States report no foreign residence history or known contact with another person who had leprosy. Therefore, they probably acquired the disease from local sources (*1*).

Leprosy is believed to be transmitted mainly from person to person through infectious aerosols or direct contact (*1*). However, there is a strong genetic component with regards to susceptibility to infection, and 95% of all persons appear to be naturally resistant to leprosy (*8*). *M. leprae* is an obligate intracellular parasite that can survive for only short periods unprotected in the natural environment (*9*), and few animals support experimental infection with this bacterium (*10*). The only known nonhuman reservoir of *M. leprae* is the nine-banded armadillo (*Dasypus novemcinctus*), and disease prevalence rates among armadillos may exceed 20% in some locales (*11*).

Armadillos are highly susceptible to *M. leprae* and can manifest massive burdens of bacilli in their tissues (10^10–11^ organisms/g). This sylvatic infection was first detected in 1975 but is known to have occurred among armadillos for many decades before that time (*12*–*14*). Early surveys in the United States suggested that leprosy was restricted mainly to armadillos in Texas and Louisiana. No evidence for infection was found among armadillos in Florida, Georgia, and Alabama (*12*,*14*,*15*). However, in recent times, the geographic range of the infection seems to be expanding (*16*).

We recently showed that armadillos over a 4-state area in the southern United States were infected with a single predominant *M. leprae* genotype strain (3I-2-v1), and we recovered this same strain from a large number of persons with leprosy in these same states. Leprosy is probably a zoonosis in the southern United States (*17*). Armadillos are common throughout the southern United States, and their geographic range extends through Latin America to northern Argentina (*18*). To better understand the geographic range of *M. leprae*–infected armadillos and the role that these animals might play in perpetuating leprosy, we surveyed armadillos for *M. leprae* and compared genoypes of *M. leprae* isolated from these animals with those from biopsy samples obtained from patients with leprosy in the southeastern United States.

## Materials and Methods

### Study Design

In an ecologic cohort study, we surveyed armadillos and patients in the southeastern United States for *M. leprae* and genotyped isolated bacilli. Patient samples were obtained from excess diagnostic materials after a category 4 exemption was granted by the institutional review board of Louisiana State University (Baton Rouge, LA, USA). Interviews with some patients were conducted by the Florida Department of Health, and some patients in Mississippi were interviewed according to a protocol approved by the institutional review board at Forrest General Hospital (Hattiesburg, MS, USA). Armadillos were collected according to established protocols approved by the Institutional Animal Care and Use Committee at the Valdosta State University (Valdosta, GA, USA) and the University of Georgia (Athens, GA, USA).

### Collection of Samples from Wild Armadillos

**Figure 1:**
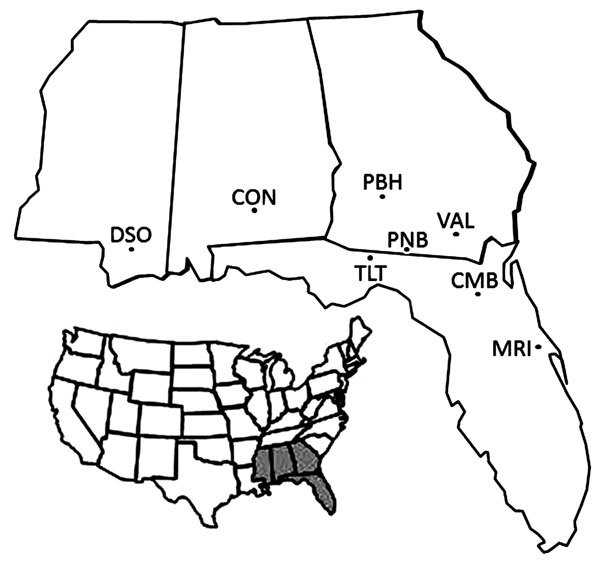
Eight locations in 4 states in the southeastern United States where armadillos were sampled and tested for infection with *Mycobacterium leprae*. Inset (shaded region) indicates location of the 4 states. DSO, DeSoto National Forest, Mississippi; CON, Conecuh National Forest, Alabama; PBH, Pebble Hill Plantation, Thomasville, Georgia; PNB, Pinebloom Plantation, Albany, Georgia; VAL, Valdosta, Georgia; TLT, Tall Timbers Research Station and Land Conservancy, Tallahassee, Florida; CMB, Camp Blanding, Florida; MRI, Merritt Island National Wildlife Refuge, Florida.

Blood and reticuloendothelial tissue samples were collected from 645 armadillos at 8 locations in state and federal Wildlife Management Areas, Forests, and Refuges in Mississippi, Alabama, Georgia, and Florida during 2003–2012 (Figure 1). Armadillo serum or whole blood samples were dried on filter paper (Nobuto strips; Advantec, Dublin, CA, USA), and tissue samples were frozen or fixed in 70% ethanol. These specimens were shipped to the National Hansen’s Disease Program (Baton Rouge, LA, USA) for testing. In addition, we reexamined 55 frozen serum samples from armadillos collected in Florida during 1983–1988 (*11*).

### Biomarkers for *M. leprae* Infection

Serologic and molecular assays were used to identify armadillos infected with *M. leprae.* Serum samples were tested for IgM against phenolic glycolipid-1 (PGL1) antigen of *M. leprae* (BEI Resources, Manassas, VA, USA) and for leprosy IDRI diagnostic-1 (LID1) antigen (Infectious Disease Research Institute, Seattle, WA, USA) by using an ELISA as described (*19*). Positive results were determined according to optical density and by using limits described for the PGL1 assay (*13*,*19*). Interpretations for the LID1 ELISA were derived by inspecting the rank-ordered distribution of optical densities for deflection from linearity, and arbitrarily assigning a value limit. DNA was extracted from lymph nodes or spleens of animals seropositive by ELISA by using the DNA Easy Kit (QIAGEN, Valencia, CA, USA) and screened by using a PCR with primers specific for regions of the *M. leprae* multicopy repeat sequence and the heat shock protein gene encoding the 18-kD antigen as described (*20*). Amplicons were confirmed by sequencing.

### Patient Samples

Skin biopsy specimens collected from patients attending the National Hansen’s Disease Program outpatient clinic or referred for diagnosis were stored frozen in optimum cutting-temperature compound or archived as formalin-fixed, paraffin-embedded blocks and occasionally fixed in 70% ethanol. To assess *M. leprae* genotype strains in the region, we used 52 biopsy specimens from cases-patients with leprosy during 2007–2012. Samples consisted of 47 fixed in formalin and embedded in paraffin, 4 fixed in ethanol, and 1 frozen.

### Genotyping of *M. leprae* from Armadillos and Patients

**Figure 2:**
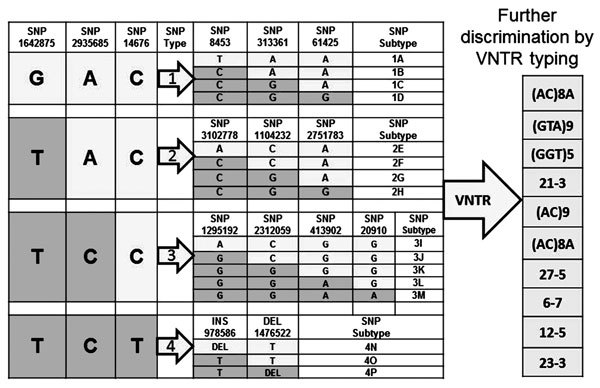
Genotyping scheme for *Mycobacterium leprae* determined by using single-nucleotide polymorphisms (SNPs) and variable number tandem repeat (VNTR) polymorphisms, southeastern United States. Shading indicates the base that differentiates SNP type and subtype of *M. leprae*. The algorithm used for strain typing of *M. leprae* is based on specific SNP location and type and VNTR copy number at the various locations identified along the chromosome. After identification of the major SNP subtype, *M. leprae* is further discriminated by using allele numbers at 10 VNTR loci. INS, insertion; DEL, deletion.

We genotyped *M. leprae* isolated from 52 patients and selected armadillo samples, and assigned their phylogenetic affiliation by using an algorithm associating 16 major single-nucleotide polymorphisms (SNPs) as described (*6*,*17*) (Figure 2). Because SNP-type 3I predominates in North America, we first sequenced SNP7614 and insertion/deletion_17915. Samples with a single copy of insertion/deletion_17915 and a T at SNP7614 were confirmed as 3I and further discriminated as 3I-1 or 3I-2 on the basis of SNP-1527056. Non-3I isolates were typed for SNPS as described (*6*,*17*).

**Figure 3:**
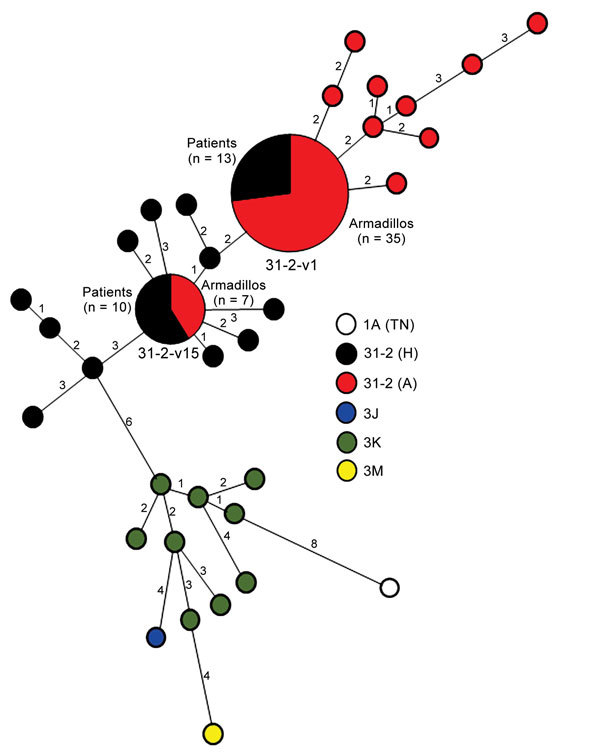
Minimum spanning tree constructed by using single-nucleotide polymorphism (SNP) and variable number tandem repeat (VNTR) polymorphism profiles for *Mycobacterium leprae* samples from patients and armadillos from the southeastern United States. Each circle represents a single strain genotype of *M. leprae.* Large circles indicate that >1 sample (number shown) had a common genotype. Values along lines indicate number of differences between allelic profiles. Human and armadillo samples of common SNP subtype 3I-2 are indicated by different colors. Only 2 genotypes were present in >1 sample, and both genotypes were present among armadillos and patients.

To enhance discrimination of isolates with an identical SNP type, we determined the copy number of 10 variable number tandem repeats (VNTRs) in a lineage dependent manner as described (*17*). Multiplex nested PCR amplified all 10 VNTR loci, and these loci were used as a template for individual assessments (Technical Appendix Table 1). VNTRs <5 bp were sequenced to determine copy number, and those >5 bp were determined by fragment analysis. A representative number of amplicons were sequenced to confirm the fragment size (Genelab, Louisiana State University School of Veterinary Medicine, Baton Rouge, LA, USA). The array of genotypes determined for patient and armadillo isolates was plotted by using minimum spanning tree analysis in BioNumerics 7.1 software (Applied Maths NV, Sint-Latem, Belgium) in a lineage-dependent manner. VNTR further discriminated the SNP lineage (Figure 3).

### Genome Sequencing

The *M. leprae* genome sequences from 4 armadillos harboring the 3I-2-v15 genotype were obtained by fragment library sequencing by using the Ion Proton System Libraries Kit (Life Technologies, Grand Island, NY, USA). DNA quality and integrity were validated by using the Agilent 2000 Bioanalyzer (Agilent Technologies, Santa Clara, CA, USA) and sequenced with an Ion PI Chip Kit v2 (Life Technologies). The sequence data were compared with the published genome of the *M. leprae* TN reference standard (*21*), and variant calls were generated by using Partek 4.0 software (Partek, St. Louis, MO, USA). Variants with frequency >90%, and a minimum 10× coverage were compared with 3I-specific variants of the armadillo-associated *M. leprae* genotype strain 3I-2-v1 (Technical Appendix Table 2) (*17*). The 13 unique variants that differentiated 3I-2-v15 from 3I-2-v1 were confirmed by direct sequencing of additional human (n = 10) and armadillo (n = 15) isolates of both strain types (primer sequences Technical Appendix Table 3).

## Results

### Expanded Geographic Range of *M. leprae* Infection among Armadillos

We screened blood and tissue samples to determine the prevalence of *M. leprae* infection among 645 armadillos obtained at 8 locations in the southeastern United States (Mississippi, Alabama, Georgia, and Florida) (Figure 1). We detected antibodies to *M. leprae*−specific antigens at each location and in 16.4% (106/645) of all the samples screened: 10.1% (65/645) had antibodies to PGL1, and 9.9% (64/645) had antibodies to LID1. Only 23 samples showed positive results in both assays. These samples included LID1 antigen−enhanced serologic detection of infection versus screening with PGL1 alone (Table 1).

**Table 1:** Serologic and molecular detection of *Mycobacterium leprae* infections among armadillos from various locations in the southeastern United States*

**Location**	**No. blood or serum samples**	**Serologic screening, no. samples positive for *M. leprae* antigen **				**No. lymph node samples tested**	**PCR, no. samples positive for *M. leprae* DNA**			
		LID1	PGL1	LID1 or PGL1	LID1 and PGL1		RLEP locus	*hsp 18* locus	1 site	2 sites
**CMB**	31	2	2	2	2	2	2	0	2	0
**VAL**	8	1	0	1	0	0	0	0	0	0
**MRI**	65	9	16	18	7	17	16	14	17	13
**CON**	38	5	2	7	0	5	5	2	5	2
**DSO**	23	7	0	7	0	7	6	3	7	3
**PNB**	117	11	13	20	4	19	19	19	19	19
**PBH**	23	5	1	6	0	3	3	2	3	2
**TLT**	340	24	31	45	10	42	41	36	42	36
**Total**	645	64	65	106	23	95	92	76	95	75

*LID, leprosy IDRI diagnostic 1 antigen; PGL, phenolic glycolipid 1 antigen; RLEP, mutlicopy repeat sequence; *hsp*, heat shock protein; CMB, Camp Blanding, FL; VLD: Valdosta, GA; MRI, Merritt Island National Wildlife Refuge, FL; CON, Conecuh National Forest, AL; DSO, DeSoto National Forest, MS; PNB, Pinebloom Plantation, Albany, GA; PBH, Pebble Hill Plantation, Thomasville GA; TLT, Tall Timbers Research Station and Land Conservancy, Tallahassee, FL.

*M. leprae* was not found among armadillos in this region before 2009 (*11*,*16*). Two of the areas surveyed (Tall Timbers Research Station and Land Conservancy, Tallahassee, Florida, and Pinebloom Plantation, Albany, Georgia) also had been sampled in earlier studies (*14*). In addition, we examined 55 serum samples collected from armadillos in nearby regions of Florida. These samples had been stored frozen since 1983–1988 (*11*). Rescreening these samples by using the current PGL1 and LID1 ELISAs, we again found no serologic reactivity, which confirmed the earlier findings.

Lymph node tissues were available from 95 of the 106 animals considered serologically positive by either ELISA. DNA was extracted from tissues and tested by PCR for *M. leprae*−specific multicopy repeat sequence and heat shock protein 18 gene fragments. All 95 samples amplified in >1 *M. leprae*−specific PCR, and 75/95 (80%) amplified with both PCRs (Table 1). Amplicon sequencing confirmed specificity for *M. leprae.*

### *M. leprae* Isolated from Armadillos and Patients

Sufficient DNA was available to genotype the *M. leprae* recovered from 42/95 armadillos and from the biopsy samples of 52 patients who had leprosy in the same geographic region. Among armadillos, only 2 *M. leprae* genotype strains were recovered. We found 35/42 (83%) of the animals harbored *M. leprae* SNP-VNTR type 3I-2-v1, which we had identified as infecting patients and armadillos in Texas, Louisiana, Mississippi, and Arkansas (*17*). Therefore, type 3I-2-v1 can be found among armadillos in Mississippi, Alabama, Georgia, and northern Florida. However, in southern Florida, we found 7 armadillos infected with an *M. leprae* genotype strain not previously observed among armadillos. Designated 3I-2-v15, this new armadillo-associated genotype strain differed from 3I-2-v1 by having multiple allele changes at 3 VNTR loci. The 3I-2-v15 allele profile was unique and had not been previously identified in a global database of *M. leprae* VNTR strain types (*22*). According to allele frequencies derived from that database, the 3I-2-v15 genotype had only a 1:3,700 probability for random recombination within any of these 7 samples. Subsequent deep sequencing of 3I-2-v15 isolates from 4 armadillos showed that this genotype was uniform and consistent among all animals examined and had multiple SNP differences between 3I-2-v15 and 3I-2-v1 *M. leprae.* Four SNPs common among 3I-2-v1 isolates were not present in 3I-2-v15, and 9 additional common SNPs were unique to 3I-2-v15 (Table 2). These same 13 polymorphisms were confirmed by direct PCR of *M. leprae* from an additional 10 human and 15 armadillo isolates. 3I-2-v15 is the most diverse representative of the 3I-2 lineage sequenced to date.

**Table 2:** Next-generation whole-genome sequencing of *Mycobacterium leprae* strain 3I-2-v15 derived from wild armadillos from the southeastern United States compared with that for armadillo-associated strain 3I-2-v1

**Sample no.**	Average coverage (% genome covered)*	**No. variants partially shared†**	**No. variants only in 3I-2-v15‡**	**No. 3I-2 variants absent in 3I-2-v15‡**	**No. variants in both strains**	**No. TN strain variants in both strains**
**US-36**	19.8 (88.72)	7	9	4	20	37
**US-95**	97.99 (98.36)	1	9	4	14	37
**FL-26**	119.32 (99.00)	0	9	4	13	37
**MRI-9**	25.3 (92.08)	6	9	4	19	37

*Average no. of consensus sequence reads obtained from the particular specimen covering the entire genome. †No. variants reported to be specific for single-nucleotide polymorphism (SNP) type 3I-2 that are present in all samples of the 3I-2-v1 genotype and that were found in some, but not all, samples of the 3I-2-v15 genotype. ‡Resequencing referenced the M. leprae TN standard genomic sequence, identified 13 variants present only in 3I-2-v1 (2 SNP, 2 insertion/deletions) or 3I-2-v15 (9 SNP), and differentiated the strains.

### Patient Samples

In contrast to SNP-VNTR analysis of *M. leprae* from armadillos, analysis of *M. leprae* from patient biopsy specimens discriminated multiple *M. leprae* genotypes. The 3I-2 lineage, which predominates in North America (*17*), was most common and found in 41 samples. The other samples had genotypes found more commonly in other parts of the world; 9 were 3K, and 1 each were 3J or 3M lineages (*6*). SNP-VNTR genotyping showed that 30 patients were infected with entirely unique *M. leprae* genotypes. However, 22 patients, as well as armadillos, had identical *M. leprae* genotypes: 12 patient biopsy samples harbored *M. leprae* type 3I-2-v1, and 10 samples harbored newly identified type 3I-2-v15. In this study, only the 2 *M. leprae* genotype strain types recovered from armadillos were present in >1 patient. Overall, 42% (22/52) of the patients were infected with *M. leprae* genotypes that were found associated with armadillos (Figure 3). All patients harboring type 3I-2-v1 had residence histories in areas of the southern United States where they may have been exposed to *M. leprae* through armadillos. All 10 patients infected with 3I-2-v15 resided and consulted physicians in southern Florida, the only region where armadillos with this same *M. leprae* genotype strain type also had been found.

None of the patients in this study reported any previous contact with another person who had leprosy. In separate studies, small groups of patients in Florida and Mississippi were interviewed about their medical history and exposure to armadillos. Only 4 of the patients in Florida interviewed could be fully typed: 3 had *M. leprae* 3I-2-v15 and 1 had 3I-2-v1. In Mississippi, all 4 patients were infected with 3I-2-v1. None of the patients interviewed in Mississippi or Florida recalled direct contact with armadillos. All patients were familiar with armadillos in their environment, and many reported gardening and other outdoor activities that might have provided some exposure to environments possibly contaminated by *M. leprae* from armadillos.

In this study, patients with no foreign residence history had 16 times greater odds of being infected with 1 of the 2 armadillo-associated *M. leprae* genotype strain types than with any other type of *M. leprae* (odds ratio 16.8, 95% CI 3.881–73.374, p<0.0001). Patients with residence histories in areas where they may have been exposed to *M. leprae* from armadillos also had 41 times greater odds of being infected with 1 of the 2 armadillo-associated types than with any other *M. leprae* genotype (odds ratio 41.3, 95% CI 2.297–742.68, p<0.0001). Although leprosy has not previously been recognized among armadillos in Florida, 16% of the animals that we studied in the region harbored *M. leprae*, and 22/52 patients that we examined also were found to be infected with 1 of the same 2 *M. leprae* genotype strain types that we recovered from armadillos in the region.

## Discussion

Leprosy appears to be an emerging infection of armadillos throughout the southeastern United States. Most armadillos are infected with a single predominant *M. leprae* strain type (3I-2-v1), which has been associated with probable zoonotic transmission of leprosy to humans (*17*). However, armadillos in southern Florida, as well as several patients from that region, are infected with a distinctly different *M. leprae* genotype strain (3I-2-v15). Armadillos must have acquired *M. leprae* from humans within the past 400 years, after the disease was introduced into the Western Hemisphere. The 3I-2-v15 strain type was not used for in vivo propagation of *M. leprae* in armadillos. With its multiple genomic polymorphisms, this train type does not appear to have evolved recently from the 3I-2-v1 strain type. Armadillos must have acquired these infections from humans who originally harbored the strains in the region, and *M. leprae* appears to have been naturally transferred to armadillos on >1 occasion and in >1 location. Interspecies transfer of *M. leprae* between humans and armadillos appears to be rare and inefficient. However, emergence of the infection among armadillos in southeastern states, which were previously believed to be free of *M. leprae*, suggests that the disease will eventually be detected among animals throughout North America, and additional *M. leprae* genotype strains might also be acquired by animals in other locations over time.

Three epidemiologic case studies in the United States (*23*–*25*) and 1 in Brazil (*26*) have implicated contact with armadillos as a risk factor for leprosy infection. Leprosy is not highly communicable, and knowledge about potential transmission of the infection through armadillos can help reduce the overall risk for disease among persons who come in contact with these animals or environments contaminated by them. *M. leprae* may be spread through direct or indirect routes, but long-term direct contact with an infectious source is believed to be the most effective means to transmit the infection (*1*). None of the patients interviewed in this study recalled any direct contact with armadillos, although they may have had indirect exposure to *M. leprae* through gardening or other outdoor activities. Because leprosy is a rare disease, any risk for infection attributable to indirect exposure to armadillos would have to be extremely low overall. Nevertheless, persons concerned about exposure to *M. leprae* from armadillos in their environment might be advised to wear gloves while gardening or use similar general hygienic practices commonly recommended for avoiding exposure to other pathogens in the environment (*27*). Physicians caring for patients with possible exposure to *M. leprae* through armadillos should retain leprosy in their differential diagnoses for cutaneous lesions, especially for patients who do not respond well to most common therapies.

The range of armadillos in the Western Hemisphere is the southern United States, Central America, and northern Argentina. Biomarkers of *M. leprae* have been reported among armadillos in Argentina, Brazil, and Colombia (*28*–*30*). However, reports of detection of the infection have been inconsistent in different locales (*14*,*31*). Disease prevalence rates among animal populations might be influenced by the season and local variations in animal density or population structure that can affect detectability of disease (*32*). Among armadillos, typically only small numbers of animals can be screened from any given location, and relatively high prevalence rates are required to reliably detect the infection. The role that armadillos might play in helping to perpetuate leprosy throughout the Western Hemisphere merits consideration.

There are currently no established laboratory tests to aid in the diagnosis of leprosy, and the disease can only be detected once persons have clinical disease. Serologic screening for PGL1 antibodies has shown only limited utility, and effective tools to aid diagnosis or monitor progress of individual infections are needed (*33*). Wild armadillos showed considerable diversity in their response to LID1 and PGL1 antigens. Use of the antigens in combination markedly enhanced serologic detection of *M. leprae* infection among armadillos, and PCR analysis of matching tissue samples showed those reactions were highly specific for *M. leprae.* For infection of armadillos initiated by intravenous administration of 1 × 10^9^ *M. leprae*, antibodies against LID1 and PGL1 become detectable only after a delay of several months, and it appears that relatively well-established infections are required before either antibody is produced (*19*). Naturally transmitted infections would involve much lower initiating doses, and the amount of bacilli required to elicit T cell−dependent IgM responses against PGL1 might be higher that needed to initiate T cell−dependent IgG responses to LID1. Trials are underway to discern the efficacy of using these antigen combinations in screening human populations, and in 1 leprosy-endemic region, LID1 antibodies appeared to be more prevalent than PGL1 antibodies (*33**−**35*).

Elimination of an infectious mycobacterium from a wildlife species is extremely difficult and costly. Authorities have struggled for decades with bovine tuberculosis in the United Kingdom and Ireland, where the badger (*Meles meles)* plays a role in spread of the disease (*36*); in New Zealand, where the opossum (*Trichosurus vulpecula*) is responsible (*37*); and, more recently, in the northern United States, where white-tailed deer (*Odocoileus virginianus*) and other cervids are involved (*38*). It is unlikely that any effort to remove armadillos from large areas would be effective, and the removal process might provide even greater risks to humans for exposure to *M. leprae* from animals. Public education about the risk for exposure to infectious agents through animals can be highly effective. The greatest potential for exposure to *M. leprae* through armadillos would probably be direct contact with the flesh of animals hunted or prepared as food. However, armadillos can also shed leprosy bacilli into the environment in bodily secretions, and bacilli might survive extracellularly in the environment for short periods, or may even be sustained within encysted amoeba or other reservoirs for 8 months (*39*). In addition, potential involvement of insects in leprosy transmission has never been fully discounted, and the role that biting insects might play in mechanically transmitting *M. leprae* between hosts also merits attention (*9*). A better understanding of the specific risk factors that might be involved in transmission of *M. leprae* between armadillos and humans is needed.

Current leprosy control efforts focus on use of multiple antimicrobial drugs to treat clinically active human cases. The decreases in global leprosy prevalence reported over the past decade seem to validate this approach because millions of persons have been cured of leprosy. However, as 1 source of infection is brought under control, other major sources might arise. Evidence is now accumulating that leprosy is a zoonosis in North America, and the infection could extend throughout the range of the armadillo. New strategies to detect leprosy and prevent its spread will be needed. Molecular genotyping of *M. leprae* enables application of modern public health principles of infectious disease control to identify sources of infection and related clusters of new cases (*40*). Insight into the dynamics of leprosy transmission in different populations will help clarify the proportional risk related to nonhuman reservoirs and could facilitate objective development of new methods to ultimately eliminate leprosy.

**Technical Appendix.** Analysis of zoonotic leprosy in the southeastern United States by multiplex nested PCR.
